# Functional results in young adults with spina bifida. A comparative study among two therapeutic periods

**DOI:** 10.1186/1743-8454-7-S1-S23

**Published:** 2010-12-15

**Authors:** Susana Rodriguez, Esther Pages, Ampar Cuxart, Jordi Iborra, Mar Melendez, Judith Sanchez-Raya

**Affiliations:** 1 Spina Bifida Unit. Rehabilitation Department. Vall d’Hebron Hospital, Barcelona, Spain

## Background

At least 75–85% of children born with Spina Bifida (SB) are expected to reach their early adult years. The growing adult SB population encounters a variety of complications including neurological, neurosurgical, cognitive, psychosocial, renal/urological, bowel management, mobility, musculoskeletal/orthopedic, sexual function, skin, and others.

## Materials and methods

We compared two cohorts of adult patients with the diagnosis of Myelomeningocele, Meningocele, and Sacral anomalies associated with Meningocele, regularly controlled in our multidisciplinary SB Unit and treated in two different therapeutical periods. The first series included patients born before 1974 whereas the patients in the second series were born between 1982 and 1989. We collected the data from the medical history, radiographic records and also performed an interview with each patient included in this study. We collected information concerning two aspects: medical and social variables. The medical data included diagnosis, functional neurological level, shunted hydrocephalus, vertebral malformations, intelligence quotient (IQ), urological and faecal incontinence, reeducation of incontinence, orthosis and type of gait. The social data included level of education, employment, marital status and driving licence. We developed a questionnaire with 15 secondary conditions associated with SB which had to be filled in order of importance by all patients with normal or borderline IQ as per Wechsler Scale.

## Results

A total of 206 patients were included in this study: 104 patients in the first series and 102 in the second one. Both series were homogeneous in relation to gender, functional neurological level and diagnosis. We found differences statistically significant in both series in relation to shunted hydrocephalus (p=0.001), spinal deformity (p=0.043), urinary re-education and collection method (p= 0.000), faecal re-education method (p=0.000), gait orthosis (p= 0.045), and educational level (p=0.000) reflecting an improvement in the second series. We found no differences in both series in relation to IQ, incontinence, ambulation, work incorporation, marital status and driving license. Regarding the personal questionnaire, there were statistically significant differences in the perception of both samples in relation to pressure ulcers (p=0.022), urinary incontinence (p=0.001), faecal incontinence (p=0.014), urinary infection (p=0.019), IQ (p=0.012), and sexual activity (p=0.000).

## Conclusions

Management of the musculoskeletal, bowel, bladder, renal, neurological systems, and other issues in SB patients have changed and improved in the last years, regarding not only diagnostic and therapeutic procedures but also a coordinated interdisciplinary team approach that has lead these patients to reach adulthood in better conditions.

